# Serum palivizumab level is associated with decreased severity of respiratory syncytial virus disease in high-risk infants

**DOI:** 10.4161/hv.29635

**Published:** 2014-11-01

**Authors:** Michael L Forbes, Veena R Kumar, Ram Yogev, Xionghua Wu, Gabriel J Robbie, Christopher S Ambrose

**Affiliations:** 1 Department of Pediatrics; Akron Children's Hospital; Akron, OH USA; 2 AstraZeneca; Gaithersburg, MD USA; 3 Ann & Robert Lurie Children's Hospital of Chicago; Chicago, IL USA; 4 Former employee of MedImmune; Gaithersburg, MD USA; 5 MedImmune; Gaithersburg, MD USA

**Keywords:** palivizumab, serum palivizumab level, respiratory syncytial virus, pediatric intensive care unit, IMpact-RSV study, RSV-related hospitalization, severe RSV disease

## Abstract

Monthly doses of palivizumab, an RSV-specific monoclonal antibody, reduce RSV-related hospitalizations (RSVH) in high-risk children; however, no specific palivizumab level has been correlated with disease severity in humans. A post hoc analysis of a previous randomized, placebo-controlled trial evaluated the relationship between serum palivizumab level at the time of RSVH and disease severity. Pediatric intensive care unit (PICU) admission was the primary severity marker. Relationships were evaluated between disease severity and gestational age, age at enrollment, age at RSVH, presence of bronchopulmonary dysplasia, sex, race, multiple birth, household smoking, daycare attendance, sibling(s), family history of atopy, duration between most recent palivizumab dose and RSVH, and palivizumab level at RSVH. Forty-two (87.5%) of 48 palivizumab recipients with RSVH had palivizumab levels drawn; 11 were admitted to the PICU. Mean palivizumab levels were lower in PICU-admitted subjects (47.2 μg/mL) vs. non-PICU subjects (98.7 μg/mL; *P* < 0.0001); there were no statistically significant differences in other variables examined. The probability of PICU admission declined with higher palivizumab levels; there were no PICU admissions with levels ≥ 92 μg/mL. In multivariate analyses, palivizumab level was the only independent predictor of PICU admission (*P* = 0.009). Palivizumab level also correlated with duration of RSVH and PICU stay, supplemental oxygen use and duration, and mechanical ventilation use and duration (*P* < 0.05). Higher palivizumab level was associated with decreased disease severity in high-risk infants with RSVH. Findings suggest that palivizumab level has clinical relevance, and adherence to timely monthly dosing may confer additional protection among high-risk children receiving palivizumab.

## Abbreviations

BPD: bronchopulmonary dysplasia
DOMV: duration of mechanical ventilation
GA: gestational age
IgG: immunoglobulin G
MV: mechanical ventilation
NA: not applicable
PICU: pediatric intensive care unit
PICU-LOS: length of stay in the PICU
ROC: receiver operating characteristic
RSV: respiratory syncytial virus
RSVH: RSV-related hospitalization
RSVH-LOS: length of stay for RSV-related hospitalization
SD: standard deviation

## Introduction

Respiratory syncytial virus (RSV) is an important pathogen of infants and young children, causing annual epidemics of bronchiolitis and pneumonia worldwide.^1,2^ By 2 y of age, almost all infants have experienced a primary RSV infection.^3^ It is estimated that RSV causes as much as 43% to 74% of bronchiolitis and 19% to 54% of pneumonia episodes during childhood.^4^ Infection of the lower respiratory tract due to RSV accounts for approximately 126 000 hospitalizations of children under 1 y of age^5^ and approximately 400 deaths per year among children younger than 4 y of age in the United States.^6,7^ The greatest morbidity occurs among children in high-risk populations: premature infants (≤35 wk gestational age [GA]), children ≤24 mo of age with chronic lung disease of prematurity (formerly referred to as bronchopulmonary dysplasia [BPD]),^8^ and children ≤24 mo of age with hemodynamically significant congenital heart disease.^9^

Early epidemiologic studies suggested that higher maternal immunoglobulin G (IgG) antibodies to RSV were protective against RSV infection in infants younger than 6 mo.^10,11^ Additional studies also demonstrated that higher IgG anti-fusion (F) protein antibody levels correlated with a decreased incidence of severe RSV infection and reinfection.^3,12^ Clinical trials of a polyclonal human antibody preparation enriched for RSV neutralizing activity demonstrated a reduction in RSV-related hospitalization (RSVH) in at-risk infants.^13,14^ Efforts to improve the RSV-specific activity of passive immunoprophylaxis led to the development of humanized monoclonal antibodies that were 50 to 100 times more potent in neutralizing the F glycoprotein; one of these antibodies, palivizumab (MedImmune), was approved in 1998 in the United States for the prevention of serious lower respiratory tract disease caused by RSV in children at high risk for RSV disease.^15^ It was not feasible to evaluate the effect of different palivizumab doses on clinical efficacy in children during early clinical studies due to the large sample size that would have been required. In preclinical trials, palivizumab concentrations >40 μg/mL were linked to significant RSV titer reduction in the lungs of cotton rats.^16^ Although the reliability of translation of cotton rat information to humans is not known, monthly intramuscular administration of palivizumab at 15 mg/kg was selected as the dose for clinical trials due to its half-life of 20^17^ to 24 d^18^ and its ability to maintain serum levels > 40 μg/mL in most subjects.^8^ A specific serum protective level in humans was not established.^17,18^

Palivizumab administration was associated with a 55% reduction in RSVH (10.6% placebo vs 4.8% palivizumab, *P* < 0.001) and a 57% reduction in RSV-related pediatric intensive care unit (PICU) admissions (3.0% vs 1.3%, *P* < 0.05) in a randomized, double-blind, placebo-controlled trial of efficacy.^8^ In that study, serum palivizumab levels were evaluated in 42 palivizumab recipients at the time of RSVH. The objective of the current analysis was to evaluate the relationship between serum palivizumab levels at the time of RSVH and disease severity.

## Materials and Methods

The IMpact-RSV study was a randomized, double-blind, placebo-controlled trial conducted at 139 centers in the United States, the United Kingdom, and Canada. During the 1996–1997 RSV season, 1502 children were enrolled; subjects were ≤6 mo of age born prematurely at ≤35 wk GA or ≤24 mo of age with BPD.^8^ Study procedures were conducted in accordance with the Declaration of Helsinki, the US Code of Federal Regulations for protection of human subjects, and Institutional Review Boards. Subjects were excluded if they required mechanical ventilation at enrollment; had congenital heart disease; had an active or recent RSV infection; received previous immune-based RSV therapy; or had known hepatic or renal dysfunction, seizure disorder, immunodeficiency, or known allergy to IgG products. They were randomly assigned 2:1 to receive 5 intramuscular injections of either 15 mg/kg palivizumab (n = 1002) or an equivalent volume of placebo (n = 500) every 30 d. The primary endpoint was hospitalization due to confirmed RSV infection. Per the protocol, serum palivizumab level was to be measured during the RSVH for all hospitalized subjects; the level was not required to be drawn on the day of hospital admission.^8^

In the current post hoc analysis, RSV disease severity and serum palivizumab level were evaluated in children receiving palivizumab who experienced RSVH. Serum concentrations of palivizumab were measured by an enzyme-linked immunosorbent assay (ELISA) performed at a central laboratory.^17^ The primary outcome variable was admission to the PICU, used as a marker of severity of disease. Secondary outcome variables included RSVH duration, PICU stay duration, use and duration of mechanical ventilation, and use and duration of supplemental oxygen; duration variables were measured in days. In the IMpact-RSV study, supplemental oxygen use during an RSVH was defined as a need for oxygen greater than the prehospitalization requirement. Independent variables included gestational age in weeks, age at enrollment in months, age at RSVH in months, the presence of BPD requiring medical treatment within 6 mo of enrollment, sex, race, multiple birth, smoker in the household, daycare attendance, sibling(s), family history of atopy (i.e., asthma, hay fever, eczema), duration between most recent palivizumab dose and RSVH, and palivizumab level at RSVH. No data were collected regarding the presence of other respiratory pathogens at the time of RSVH or the time interval between onset of respiratory symptoms and RSVH. Subjects were divided into those with (group 1) and without (group 2) PICU admission. All candidate independent variables were compared between the 2 groups. Results are reported as group 1 (PICU) vs. group 2 (non-PICU), mean (standard deviation [SD]) for continuous variables, and percentage for categorical variables. The statistical comparisons between group 1 and group 2 were conducted using two-sample *t* test for continuous variables. Fisher exact test was used to analyze proportions. Multivariate regression was used to identify variables independently associated with the outcome variables using stepwise selection. The associations between outcome variables and independent variables were analyzed using logistic regression for binary dependent variables (yes or no) and linear regression for continuous dependent variables (i.e., duration variables). The magnitude of the independent variable coefficient corresponds to the relative impact on the dependent outcome. Age at RSVH was grouped as 0 to <3, 3 to <6, 6 to <12, and ≥12 mo for the model. Due to dependence between age at enrollment and age at RSVH, age at enrollment was not entered into the regression model. A receiver operating characteristic (ROC) curve was derived for the relationship between palivizumab level and PICU admission. The odds ratio for PICU admission was based on the ROC-derived optimum palivizumab level threshold. Statistical significance was defined as *P* < 0.05. Statistical analysis was performed using MedCalc Statistical Software^19^ version 12.5 and SAS version 8.2 (SAS Institute).

## Results

**Figure 1.**
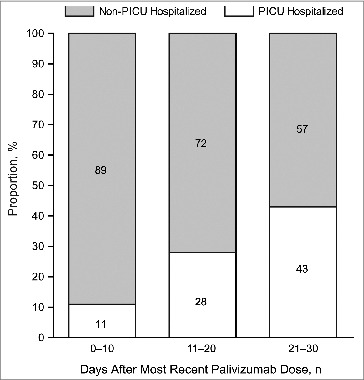
Proportion of subjects (n = 48) with PICU vs non-PICU admission by number of days post most recent palivizumab dose. PICU = pediatric intensive care unit.

Of the 1002 subjects who received palivizumab, 48 were hospitalized due to laboratory-confirmed RSV lower respiratory tract infection. All 48 subjects were ≤35 wk GA (39 with BPD). Among these 48, 13 (27%) were admitted to the PICU for a mean (SD) of 10.8 (10.6) days; 7 required mechanical ventilation for a mean (SD) of 12.4 (7.5) days. Although not statistically significant, PICU subjects had a longer time interval between receipt of their most recent palivizumab dose and RSVH than non-PICU subjects (mean [SD]): 19.2 (8.3) days vs 14.4 (10.1) days, *P* = 0.14. The proportion of subjects hospitalized by time interval is shown in **Figure 1**. There was a nonsignificant trend of increased PICU stay duration (*P* = 0.07) and MV duration (*P* = 0.07) as the duration between the most recent palivizumab dose and RSVH increased.

Forty-two of the 48 (87.5%) had serum palivizumab levels tested and available for analysis. Clinical characteristics of the PICU (n = 11) and non-PICU (n = 31) subjects with palivizumab levels as well as the 6 subjects not tested for palivizumab levels are depicted in **Table 1**. The clinical characteristics of subjects not tested for palivizumab levels were similar to those of the 42 subjects with palivizumab levels. Palivizumab levels were drawn on days 0–5 (mean, 1.9 d) of RSVH for PICU subjects and days 0–7 (mean, 1.6 d) for non-PICU subjects; overall, 90.5% (38/42) of palivizumab levels were obtained by day 3 of RSVH. The timing of RSVH by calendar month was similar in both groups. Mean (SD) palivizumab level was lower in PICU-admitted subjects compared with non-PICU subjects (47.2 [22.6] μg/mL vs 98.7 [52.4] μg/mL, *P* < 0.0001); results were similar when limited to subjects with BPD (47.2 [22.6] μg/mL vs 103.9 [55.5] μg/mL, *P* = 0.0002). No differences were observed in the other independent variables.

**Table 1.** Clinical characteristics

	Subjects with Serum Palivizumab Level		
Variable	PICU Subjects (N = 11)	Non-PICU Subjects (N = 31)	Subjects Not Tested for Serum Palivizumab Level (N = 6)
Age at Enrollment (months), Mean (SD)	7.6 (5.8)	5.6 (3.1)	5.8 (3.9)
Male, n (%)	6 (55%)	15 (48%)	4 (67%)
White/Non-Hispanic, n (%)	5 (45%)	15 (48%)	4 (67%)
BPD, n (%)	11 (100%)	23 (74%)	5 (83%)
Gestational Age (weeks), Mean (SD)	26.6 (2.0)	27.5 (3.1)	28.3 (2.5)
Age at RSVH (months), Mean (SD)	9.6 (5.8)	7.4 (3.2)	7.8 (4.2)
Multiple Birth, n (%)	5 (45%)	9 (29%)	0
Days between RSVH and Most Recent Dose, Mean (SD)	18.5 (8.7)	14.1 (9.9)	18.7 (11.2)
Smoker in Household, n (%)	6 (55%)	17 (55%)	3 (50%)
Daycare Attendance, n (%)	0	2 (6%)	1 (17%)
Has Sibling, n (%)	9 (82%)	21 (68%)	2 (33%)
Family History of Atopy, n (%)	6 (55%)	16 (52%)	3 (50%)
^a^Palivizumab Level (μg/mL), Mean (SD)	47.2 (22.6)	98.7 (52.4)	NA
^a^RSVH-LOS (days), Mean (SD)	18.5 (11.3)	4.9 (3.8)	6.0 (3.4)
^a^Supplemental Oxygen Use, n (%)	11 (100%)	17 (55%)	5 (83%)
^a^Duration of Supplemental Oxygen (days), Mean (SD)	16.3 (9.9)	3.1 (4.0)	5.0 (3.5)
PICU-LOS (days), Mean (SD)	12.0 (11.1)	NA	1.3 (2.1)
MV Use, n (%)	7 (64%)	NA	0
DOMV (days), Mean (SD)	7.9 (8.5)	NA	0

Values are given as mean value (SD), where applicable.

^a^*P* <0.01 for comparisons between PICU and non-PICU groups using two-sample t-test and Fisher exact test for continuous and categorical variables, respectively. Abbreviations: BPD, bronchopulmonary dysplasia; DOMV, duration of MV; RSVH-LOS, length of stay for RSV-related hospitalization; PICU-LOS, length of stay in the PICU; MV, mechanical ventilation; NA, not applicable; PICU, pediatric intensive care unit; RSVH, RSV-related hospitalization; RSV, respiratory syncytial virus.

**Figure 2.**
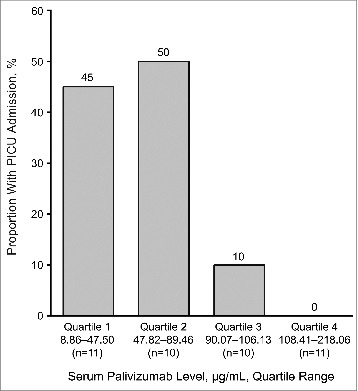
Proportion of subjects (n = 42) with PICU admission by serum palivizumab level quartile. PICU = pediatric intensive care unit. The quartiles were determined by arranging the palivizumab levels from lowest to highest; the cutoff value for each quartile was calculated using MedCalc Statistical Software.^19^

In an analysis of the correlation by serum palivizumab level quartile (determined by arranging the palivizumab levels from lowest to highest; the cutoff value for each quartile was calculated using MedCalc Statistical Software),^19^ a concentration response relationship was observed (**Fig. 2**). There were no PICU admissions when palivizumab level was ≥92 μg/mL. The inverse association between palivizumab level and PICU admission risk remained significant after multivariate regression (OR, 0.97; *P* = 0.009). Controlling for other factors, for each 10 μg/mL increase in palivizumab level, the odds of PICU admission declined by 27% (*P* = 0.009). Secondary markers of disease severity were also evaluated as outcome variables by multivariate regression. Controlling for other factors, for each 10 μg/mL increase in palivizumab level, the duration of RSVH decreased by 0.7 d (*P* = 0.002), duration of PICU stay decreased by 0.6 d (*P* = 0.003), odds of supplemental oxygen use decreased by 30% (*P* = 0.008), duration of supplemental oxygen decreased by 0.8 d (*P* < 0.001), odds of mechanical ventilation use declined by 25% (*P* = 0.03), and mechanical ventilation duration decreased by 0.4 d (*P* = 0.007).

**Figure 3.**
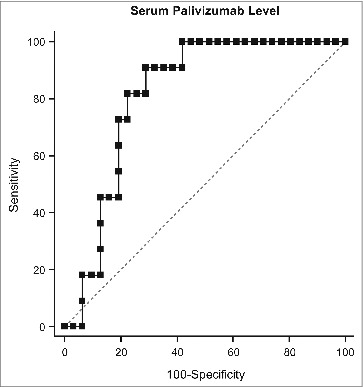
The receiver operating characteristic plot of serum palivizumab level and PICU admission. PICU = pediatric intensive care unit.

ROC analysis (**Fig. 3**) confirmed the ability of palivizumab level to discriminate between those subjects with and without a PICU admission, with a C-statistic of 0.815 (0.665–0.918, *P* < 0.0001). At the palivizumab level of 60 μg/mL, sensitivity was 81.8% and specificity was 77.4%; at 70 μg/mL, sensitivity was 90% and specificity was 70%. Subjects with palivizumab levels below 60 μg/mL were more likely to be admitted to the PICU (OR = 15.43, 95% CI: 2.69–88.63).

## Discussion

Although randomized placebo-controlled trials have demonstrated that palivizumab administration reduces RSVH in high-risk children,^8,9^ these are the first clinical data to describe the correlation between the observed serum palivizumab level and RSV disease severity in children receiving palivizumab. Higher palivizumab level was associated with decreased severity of RSV disease in high-risk infants as measured by PICU admission, duration of RSVH stay, duration of PICU stay, and supplemental oxygen use and duration. This analysis suggests that serum palivizumab level has clinical relevance and reinforces current efforts to emphasize the importance of maintaining adequate palivizumab level, for example, through timely monthly dosing with the current approved regimen of 15 mg/kg. Palivizumab levels higher than the commonly believed target level of 40 μg/mL may confer additional protection among high-risk children.

Because palivizumab levels were only available for children who experienced RSVH, the association between palivizumab level and the incidence of RSVH could not be evaluated. However, among children with RSVH, a robust association with disease severity was observed even after controlling for other factors, including the presence of BPD. Before this analysis, the only available data regarding the relationship between palivizumab level and RSV protection were derived from the cotton rat model, which demonstrated a 100-fold (2 log_10_) reduction of lung RSV titers in all animals with serum concentrations > 40 μg/mL.^16^ As a result, it has been suggested that this represents a “protective concentration” in children receiving palivizumab^20,21^; however, this conclusion is not supported by clinical data. Even among the cotton rats studied, 40 μg/mL did not represent a threshold palivizumab concentration; additional reductions in viral titer were observed at higher serum concentrations.^22^ Of interest, another monoclonal antibody against the F protein (RSHZ19) was as effective as palivizumab in the cotton rat model but failed to protect infants using a dose of 10 mg/kg that achieved serum levels that reduced the pulmonary RSV load in animals by 99%.^23^ The failure of that antibody further supports our finding that even if levels comparable to those in the cotton rat are achieved, it is not sufficient. Additionally, there is significant interpatient variability in palivizumab levels; in clinical studies, 25% of infants receiving the recommended dose (i.e., 15 mg/kg every 30 d) had serum trough concentrations <40 μg/mL after 4 monthly doses.^24^

Palivizumab levels will be lower if dosing every 28–30 d is delayed due to logistical or scheduling issues. The current data suggest that lower serum palivizumab concentrations consequent to variability in levels among patients given the 15 mg/kg dose or giving a reduced dose could increase the patient's risk of severe RSV disease. Although the palivizumab level associated with protection from RSVH has not been established, the ROC analysis demonstrated that subjects with palivizumab levels below 60 μg/mL were more likely to be admitted to the PICU; there were no PICU admissions with levels ≥92 μg/mL. This study suggests that in this cohort levels up to 92 μg/mL provide additional protection against severe RSV disease. Further prospective validation of this statistically significant association would require measurements of palivizumab level at the time of RSVH. These data also suggest that the efficacy of palivizumab may be higher if administered at doses greater than 15 mg/kg, especially in children with BPD. Further studies are needed to investigate this possibility.

The magnitude of interpatient variability in serum levels of palivizumab found in the current study are typical of other monoclonal antibodies that follow linear pharmacokinetics. Extensive pharmacokinetic modeling performed on observed serum palivizumab levels from 13 pediatric studies did not identify any factor other than body weight as a contributor to variability in palivizumab levels.^25^ Given the mean half-life of palivizumab of 20 d, with interpatient variability in the rate of decline,^17^ it is not surprising that serum levels decline during the weeks following dosing. RSV disease severity appeared to increase in proportion to the number of days since the subjects’ last palivizumab dose. This observation requires prospective validation studies. This finding also buttresses the premise that adequate palivizumab levels reduce RSV disease severity, rather than the severity of the disease somehow inducing a decrease in the palivizumab level. This conclusion is supported by reports of increased rates of RSVH associated with incomplete compliance^26^ and epidemiologic studies that demonstrated decreased incidence of severe RSV infection in children with higher IgG anti-fusion (F) protein antibody levels.^3,12^

The primary limitation of this retrospective analysis is the small sample of RSVH among palivizumab recipients. This is due, in part, to the efficacy of palivizumab in reducing RSVH. However, despite the small sample, the correlations observed were statistically significant. Palivizumab levels were not tested for all subjects with RSVH; however, the proportion of children not tested was low (12.5%) and these subjects’ clinical characteristics were similar to those of the 42 evaluable subjects. Palivizumab concentrations at the time of RSV infection were not available. However, given the half-life of palivizumab and the fact that levels were measured within 3 d of RSVH in 90% of the subjects, the measured concentrations were likely similar to those present at the time of RSV infection. Additionally, although multivariate analysis controlled for several potential covariates, it is possible that the association between palivizumab level and RSV-related PICU admission could be explained by an unknown confounder. Given the strength and biologic plausibility of the association observed, this appears unlikely. Although the analyzed data are from a study conducted in 1996–1997, PICU admission and other markers of disease severity are still relevant to current pediatric practice; even if PICU admission criteria have changed, the data set remains internally consistent and valid. While there are many factors that could influence why and for how long a child was admitted to the PICU (e.g., bed availability, hospital practice), such parameters were not collected in the IMpact-RSV study; furthermore, one would not expect these factors to be associated with palivizumab level. In addition, the broad range of countries and clinical practices that participated in the IMpact-RSV study reduces the likelihood that PICU admissions were a function of bed availability or other system factors. The results of this study suggest that serum palivizumab levels are a significant predictor of RSV disease severity and PICU admission, reinforcing the need for sustaining palivizumab levels through timely monthly dosing with the current approved regimen of 15 mg/kg.
